# Post-COVID psychiatry functional unit of hospital del mar: Treatment and monitoring of COVID-19 patients who developed neuropsychiatric symptoms

**DOI:** 10.1192/j.eurpsy.2023.879

**Published:** 2023-07-19

**Authors:** L. Vargas Puértolas, S. Oller Canet, E. Pechuán Martínez, À. Arroyo Núñez, T. Legido Gil

**Affiliations:** 1 Psychiatry; 2 Mental health nurse; 3 Psychology, Hospital del Mar, Barcelona, Spain

## Abstract

**Introduction:**

There is growing evidence on the prevalence of neuropsychiatric disorders in patients suffering from COVID-19. On Hospital del Mar, a multidisciplinary post-COVID unit was created in May 2020 for COVID-19 patients.

**Objectives:**

Explain the functioning of the Post-COVID Psychiatry Functional Unit and the sociodemographic and clinical characteristics of treated patients.

**Methods:**

The Post-COVID Unit assessed all patients treated for COVID at Hospital del Mar. Patients referred by their primary care center for persistent post-COVID symptoms were also treated.

During a telephone interview, they received the PHQ-4 questionnaire to identify psychiatric symptoms. If the score was => 3, the patient was referred to the Post-COVID Psychiatry Unit.

Initial contact was established by a mental health nurse, who collected a clinical history and administered three scales: PHQ-9, GAD-7 and PCL-5. A descriptive sociodemographic and clinical data analysis of patients treated by the Post-COVID Psychiatry Unit is carried out.

**Results:**

The sample consists of 149 patients who have been treated for positive PHQ-4 from July 2020 to April 2021. The majority were women (71%) and averaged 50.2 years old (SD = 12.3). In terms of the severity of COVID-19 infection, 45.6% needed hospitalization.

The mean score of the psychiatric symptom scales was 11.12 points on the PHQ-9 (SD=5.4) (1-4: minimal depression, 5-9: mild, 10-14: moderate, 15.19: moderately severe, 20-27: severe), 9.43 (SD=5.1) points in the GAD7 (cutoff point>=10) and 2.99 (SD=4) in the PCL5 (cutoff point>6).

39.5% patients of the sample were visited by psychiatry/psychology, of these 33.6% met psychiatric diagnostic criteria, the most frequent being an Adjustment disorder (15.40%), followed by Major Depressive Disorder (8.7%), Anxiety Disorder (4.7%) and other diagnoses (4.7%).

**Conclusions:**

Patients treated in the Post-COVID Unit were mainly middle-aged women, the most common symptomatology being mild depressive symptoms.

**Disclosure of Interest:**

None Declared

